# Systematic evaluation of supervised machine learning for sample origin prediction using metagenomic sequencing data

**DOI:** 10.1186/s13062-020-00287-y

**Published:** 2020-12-10

**Authors:** Julie Chih-yu Chen, Andrea D. Tyler

**Affiliations:** National Microbiology Laboratory, Public Health Agency of Canada, 1015 Arlington Street, Winnipeg, Manitoba R3E 3R2 Canada

**Keywords:** Machine learning, Metagenomics, Microbiome, Multivariate regression, Lasso regularization, Multiclass classification, CAMDA, MetaSUB

## Abstract

**Background:**

The advent of metagenomic sequencing provides microbial abundance patterns that can be leveraged for sample origin prediction. Supervised machine learning classification approaches have been reported to predict sample origin accurately when the origin has been previously sampled. Using metagenomic datasets provided by the 2019 CAMDA challenge, we evaluated the influence of variable technical, analytical and machine learning approaches for result interpretation and novel source prediction.

**Results:**

Comparison between 16S rRNA amplicon and shotgun sequencing approaches as well as metagenomic analytical tools showed differences in normalized microbial abundance, especially for organisms present at low abundance. Shotgun sequence data analyzed using Kraken2 and Bracken, for taxonomic annotation, had higher detection sensitivity. As classification models are limited to labeling pre-trained origins, we took an alternative approach using Lasso-regularized multivariate regression to predict geographic coordinates for comparison. In both models, the prediction errors were much higher in Leave-1-city-out than in 10-fold cross validation, of which the former realistically forecasted the increased difficulty in accurately predicting samples from new origins. This challenge was further confirmed when applying the model to a set of samples obtained from new origins. Overall, the prediction performance of the regression and classification models, as measured by mean squared error, were comparable on mystery samples. Due to higher prediction error rates for samples from new origins, we provided an additional strategy based on prediction ambiguity to infer whether a sample is from a new origin. Lastly, we report increased prediction error when data from different sequencing protocols were included as training data.

**Conclusions:**

Herein, we highlight the capacity of predicting sample origin accurately with pre-trained origins and the challenge of predicting new origins through both regression and classification models. Overall, this work provides a summary of the impact of sequencing technique, protocol, taxonomic analytical approaches, and machine learning approaches on the use of metagenomics for prediction of sample origin.

**Supplementary Information:**

The online version contains supplementary material available at 10.1186/s13062-020-00287-y.

## Background

Microbiome studies have demonstrated successes in detecting microbial compositional patterns in health and environmental contexts. Large scale studies which exemplify global efforts to facilitate the understanding of microbial presence and abundance in relation to diseases or environmental factors, have included the Human Microbiome Project [1], the Metagenomics & Metadesign of Subways & Urban Biomes (MetaSUB) [2] and the Earth Microbiome Project [3]. Technological advances which have enhanced the ability to both detect species and estimate their abundance in samples, include the 16S ribosomal RNA (rRNA) amplicon sequencing approach which targets and specifically sequences a region within the 16S rRNA gene of bacteria and archaea; and the shotgun whole genome sequencing approach in which all genetic material present in a sample is sequenced. The latter has the potential to allow identification of all manner of species to the strain level as well as allowing for the detection and characterization of functional units such as genes, plasmids, or pathogenicity islands. Furthermore, the concordance between the two methods is an area of active debate, with discrepancy among studies in which direct comparison of these two methods has been undertaken [4–7]. Despite the pros and cons of each technique, successes in extracting meaningful biological information have been found for disease and environmental studies using both methods [2, 3, 8–11].

The majority of analytical approaches for sample source prediction used to date have focused on supervised classification methods such as support vector machines and random forest, in order to assign trained source labels to unknown samples [9, 10, 12, 13]. Delgado-Baquerizo et al. found high variability in relative abundance across various geographical locations through examining soil microbiome, and used random forest modeling to predict habitat preference for dominant phylotypes [9]. In the Earth Microbiome Project, random forest models were built to distinguish samples from various environmental factors including association with plants or animals as well as saline presence [10]. From the perspective of identifying potentially mixed sources, SourceTracker [14] uses a Bayesian approach to estimate the proportions of source environments in a sample without the assumption of one source label. In the 2018 Critical Assessment of Massive Data Analysis (CAMDA) challenge, supervised classification approaches have been applied to predict sample source using urban microbiome with high accuracies up to 0.91, where the independent sample set was of the same origins as samples previously trained [12, 15–17].

The objective of the 2019 CAMDA metagenomics forensic challenge was to use urban microbiome data to predict locations of samples from new origins that had not been previously sampled (Figure S1). As classification models are limited to assigning new samples to those pre-trained origins from which some samples were already collected and trained, they can never predict a novel origin. Thus, for the purpose of predicting new origins, an alternative approach must be used. One such method is to model urban origins using geographic coordinates, as was demonstrated in a report on the association between human genetics and geographical locations [18]. While the evolution of microorganisms with respect to locations can be quite different, the association between latitude and microbial composition in various contexts has been described in several studies [19–22]. Richness and diversity in planktonic marine bacteria, and the surface microbiome from ambulances in the United States were found to be inversely correlated with latitude, a pattern called the “latitudinal diversity gradient” [20, 21]. In the gut microbiome, Suzuki et al. found significant positive and negative correlations to latitude with Firmicutes and Bacteriodetes, respectively in 23 populations, using 16S-based data [22], while Fisman et al. reported correlation between bloodstream infection from gram negative bacteria and proximity to the equator measured by latitude-squared [19]. Such patterned variability may be used to assist in prediction of novel origins, and was investigated for this study.

Given the availability of both 16S rRNA amplicon and shotgun data, we first set out to compare and contrast normalized organism abundance from datasets generated using 16S amplicon and shotgun sequencing technologies in a dataset derived from a single location (Boston). We then used the knowledge obtained from this analysis to perform sample source attribution to a new geographic origin, modeling the longitude and latitude as outcome variables. The normalized taxonomic abundance levels were used as features, and the multivariate regression model with Lasso regularization was selected for prediction of new sample origins to avoid model overfitting. Subsequently, we compare prediction performance between multivariate regression and multiclass classification models for the mystery data from new origins. Lastly, we report a computational approach to identify whether a sample is from a new or pre-trained origin through the Simpson’s diversity index on classification probabilities.

## Methods

Analyses were conducted in R 3.6.0 version unless otherwise stated.

### Dataset description

All Boston Urban [11] and MetaSUB [2] datasets were provided by the CAMDA organizers: 1) Boston pilot 16S and shotgun data of 23 samples, 2) MetaSUB shotgun data of 294 samples from 16 cities for training and 3) MetaSUB shotgun data of 60 mystery samples from 8 new city origins as the independent test set. For the Boston dataset, sequencing of the V4 hypervariable region of the 16S rRNA gene was conducted as described by Hsu et al. [11]. Both 16S and shotgun abundance tables (denoted as 16S and SG-MP) corresponding to these samples were generated using QIIME v1.8 [23] and MetaPhlAn2 [24], respectively [11]. We further analyzed the Boston shotgun data using the Kraken2-Bracken [25, 26] assignment approach described below (denoted as SG-KB). For the machine learning models, the downloaded MetaSUB shotgun training dataset included sequencing data from 16 cities: Auckland (AKL), Berlin (BER), Bogota (BOG), Hamilton (HAM), Hong Kong (HGK), Ilorin (ILR), London (LON), Marseille (MAR), New York (NYC), Offa (OFA), Porto (PXO), Sacramento (SAC), Sao Paulo (SAO), Sofia (SOF), Stockholm (STO) and Tokyo (TOK). Fifteen of the training cities (276 samples) had paired-end sequencing data at 150-basepair (bp) reads, whereas SAC data were single-ended with 125 bp reads. Additional information on samples regarding sequence lengths, single−/paired-end and percentage of unclassified reads were reported in Table S1.

### Taxonomic abundance estimation and data processing

Taxonomic sequence classification and organism abundance estimation for shotgun datasets were conducted using Kraken2 [25] and Bracken [26], respectively, using a customized reference database which included reference sequence representatives of the bacterial, viral, archaeal groups as well as the human genome, all obtained from NCBI Refseq release 91 on December, 2018. The Bracken database was constructed using 150 bp as the read length, since these were the most commonly identified in the CAMDA dataset. Reads assigned to the human genome were filtered out. Normalization of the total abundance count table for each taxonomic scale was done using the cumulative sum scaling (CSS) approach at the 50th percentile (metagenomeSeq [27] R package). The CSS normalization corrects for library size differences by scaling based only on the lower abundant taxa under the 50th percentile, so the normalized abundance is different from the standard relative abundance that have the same total sum from all taxa. Hence we refer to this dataset as normalized abundance. To avoid spurious results from sparse features, taxa that did not contain at least 100 reads in more than one and eight samples were filtered out in the Boston and MetaSUB datasets, respectively. The Bray Curtis dissimilarity and principal coordinate analysis with the Cailliez correction for negative eigenvalues were powered by the vegan [28] and ape [29] R packages.

### Machine learning models for prediction of sample source

Instead of estimating latitude and longitude in separate models, we chose to take into account dependencies between the two coordinates, and modeled them together using multivariate regression with Lasso regularization (glmnet R package) [30]. For each taxonomic model, the normalized and log_2_-transformed microbial abundance was standardized as input features, and latitudes and longitudes were the response variables. Ten-fold cross validation was conducted with mean squared error (MSE) evaluation to choose the hyperparameter λ such that the error from the model is within one standard error of the minimum. The MSE reports the average squared differences between the actual and estimated values from a model. While there are other metrics and debates on the comparison to mean absolute error [31, 32], MSE was used for the assessment of models. Performance was reported using 10-fold nested cross validation (CV) to evaluate prediction accuracy of samples from pre-trained cities, whereas leave-1-city-out (L1CO) CV was used to evaluate accuracy of samples from new cities. For comparison, a classification (Lasso-regularized multinomial logistic regression) approach was also performed using the glmnet R package [30]. Due to the sample size imbalance between cities, samples were weighted when training the classification model. The weights were calculated as 1 – (sample count of the corresponding city / total sample count). While accuracy is a common performance measure for classification models, MSE was also reported for prediction on mystery samples using the classification model in order to compare to the regression model. Lastly, as the logistic regression is based on the assumption of linearity between log odds and the features; we also performed the random forest classification with down-sampling using the randomforest R package, and reported the performance.

### Binary machine learning classifier on prediction ambiguity

To inform whether a sample is from a pre-trained city, a Simpson’s diversity value, which is the equivalent of the Gini impurity for decision trees, can be computed using class prediction probabilities of each sample from the sample source Lasso-regularized classification model (vegan R package [28]). The index was used to reflect the prediction ambiguity of each sample from the model, as was previously proposed in a genomic study [33]. Samples with higher prediction ambiguity are expected to have prediction probabilities distributed across multiple classes, as reflected by higher diversity index in class probabilities. Overall, two diversity values were obtained for each sample in L1CO and 10-fold CV settings, mimicking the predictions of a new origin and a pre-trained origin, respectively. Subsequently, a Bayes classifier (naivebayes R package [34]) using kernel density estimation was built to learn the two distributions of diversity values from L1CO CV versus 10-fold CV. The learned model maximized the probability of assigning the correct class (L1CO or 10-fold CV) of training samples, given the Simpson’s diversity values as the sole feature. Classifier performance was evaluated by leaving each Simpson’s value out and predicting whether it was from the L1CO or 10-fold CV setting. The final classifier was then used to predict whether a mystery sample is from a new origin, given the impurity of its source prediction probabilities.

## Results

### Abundance differences between technological and analytical approaches

**Fig. 1:**
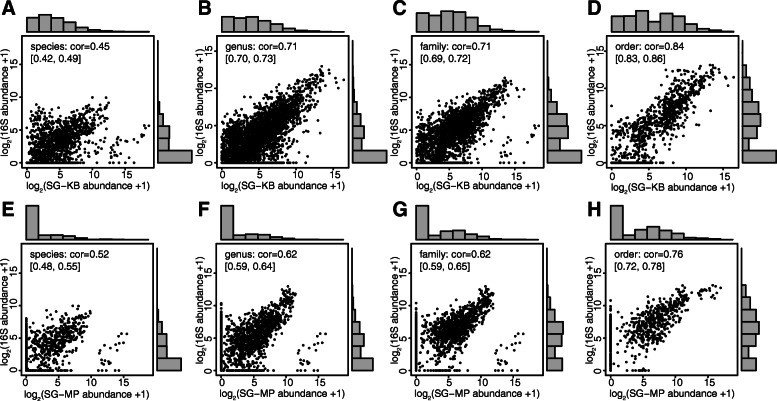
Normalized and Log_2_-transformed abundance between Boston Pilot’s 16S and shotgun data at different taxonomic scales. The y-axes represent the abundance from 16S amplicon data. **a**-**d** The x-axes represent the abundance extracted using Kraken2 and Bracken from shotgun data (SG-KB). **e**-**h** The x-axes represent the abundance extracted using MetaPhlAn2 from shotgun data (SG-MP). Histograms on top and right-hand panels show the distributions of shotgun data and 16S data, respectively. Correlations were reported using normalized but non-transformed abundance, followed by the corresponding 95% confidence intervals [CI]

**Fig. 2:**
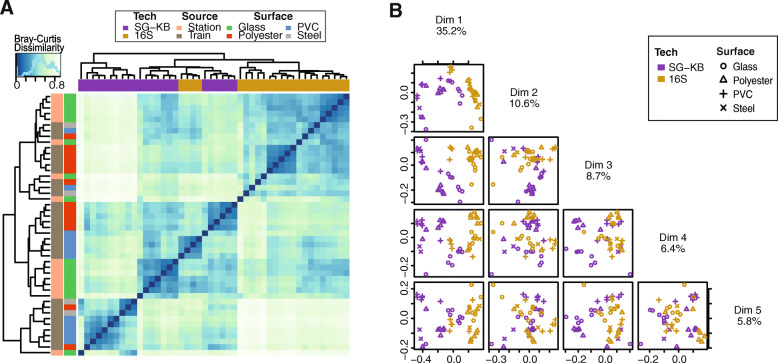
Relations between 16S rRNA amplicon and shotgun sequencing data generated from the same Boston samples. **a** Heatmap visualization of the Bray-Curtis dissimilarity matrix computed from Genus abundance of 16S and shotgun (SG-KB) data **b**The first five dimensions of PCoA computed using Bray-Curtis dissimilarity

Given that both 16S and shotgun approaches were used to sequence the 23 Boston samples collected from several surfaces and two sources [11], we first investigated counts of detected organisms at varying taxonomic scales and their normalized abundance using both datasets. For the shotgun data, we evaluated the abundance table extracted from Kraken2 [25] and Bracken [26] (SG-KB) as well as the table provided by CAMDA using MetaPhlAn2 [24] (SG-MP). Using the SG-KB data, we evaluated the read count thresholds of each species to inform our cutoff for taxa filtering (Figure S2). We chose a cutoff of 100 reads in consideration of the tradeoff between higher stringency and steady correlation levels with the 16S data. Overall, there is higher sensitivity in the SG-KB data at bacterial species, genus and family levels compared to the other methods. SG-MP data analysis reported the least distinct taxa at all taxonomic levels except for species (Table 1). As expected, both shotgun datasets identified more distinct species than 16S data. While there were 75 overlapping species identified between SG-KB and 16S data, there were 61 between SG-MP and 16S datasets. We next examined reported normalized abundance of commonly detected organisms between technologies in all 23 Boston samples. Pearson correlation coefficients between normalized 16S and SG-KB abundance at species, genus, family, order and class levels were 0.46, 0.71, 0.71, 0.84 and 0.89, respectively (Fig. 1a-d). The correlation coefficients between 16S and SG-KB were slightly higher than those between 16S and SG-MP data, except at the species level (One-sided paired t-test *p* = 0.0639; Fig. 1e-h). Higher correlation was found at higher taxonomic ranks. At any given taxonomic rank, variation was greater for organisms detected at lower abundance. The histograms further revealed an overall trend of inflated zero counts in 16S datasets for many taxa that were detected at low normalized abundance by SG-KB (Fig. 1a-d). On the other hand, such zero inflation was revealed in SG-MP instead when compared with the 16S data (Fig. 1f-h). The discrepancy in zero inflation between Kraken2 + Bracken and MetaPhlAn2 processed shotgun data with respect to 16S data highlights the impact on overall model interpretation due to the taxonomic identification tools and databases used. Given the higher sensitivity of SG-KB data, we proceeded using the taxonomic analysis of shotgun data using Kraken2 and Bracken for the rest of the manuscript. Examination of Bray-Curtis dissimilarities between samples from both 16S and SG-KB data showed clustering according to sequencing methods (Fig. 2a). The principal coordinate analysis (PCoA) highlighted the differences in the two sets in the first dimension, which explains 35% of the total variance, whereas samples collected from different surfaces were observed to cluster in later dimensions (Fig. 2b).

**Table 1:** Counts of unique taxa identified using Boston pilot 16S amplicon and shotgun metagenomics datasets in at least one samples. Percent overlaps from the shotgun perspective are reported in parentheses

		Overall Taxa Counts					
Technology (tool)	Taxa	species	genus	family	order	class	phylum
**Amplicon 16S**	**Bacteria**	143	328	173	83	52	18
**Shotgun (Kraken2 + Bracken)**	**All**	1630	523	201	89	37	20
	**Bacteria Only**	1516	500	186	81	33	16
	**Overlap**	75 (5%)	197 (39%)	117 (63%)	46 (57%)	23 (70%)	12 (75%)
**Shotgun (MetaPhlAn2)**	**All**	342	239	116	50	28	16
	**Bacteria Only**	322	211	102	44	23	12
	**Overlap**	61 (19%)	128 (61%)	84 (82%)	36 (82%)	17 (74%)	9 (75%)

### Modelling geographic coordinates using microbiome data

**Fig. 3:**
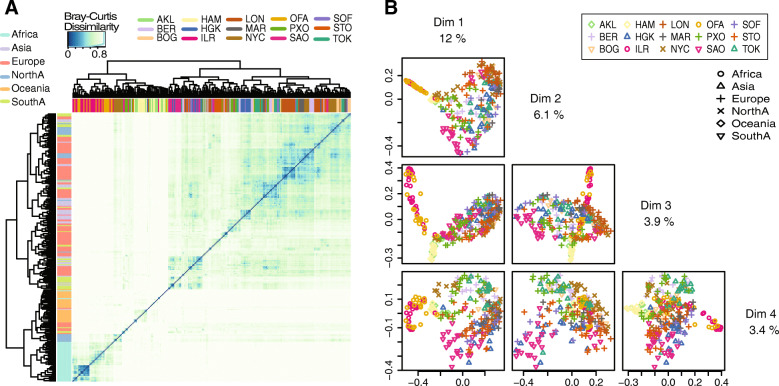
Relations between MetaSUB samples according to species abundance data. **a** Heatmap visualization of the Bray-Curtis dissimilarity matrix. The column bar colors denote cities and the row bar colors denote continents. **b** The first four dimension of PCoA using Bray-Curtis dissimilarity

**Fig. 4:**
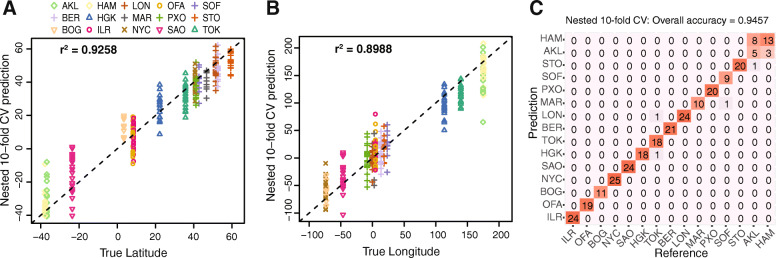
Nested 10-fold cross validation predictions from Lasso-regularized regression and classification compared to true locations. Latitude (**a**) and longitude (**b**) predictions from the multivariate regression model on species abundance data are plotted against the true geographic coordinates on the x-axis. Each data point represents a sample from the corresponding city, as indicated in the legend. The dashed line shows where predictions would be exactly correct. **c** Predictions from the classification model are illustrated in comparison to true sources. Each entry shows the number of samples predicted to be the corresponding city (row) and originally from the corresponding reference (column)

To tackle the challenge of predicting sample source in general, we first examined the training dataset of MetaSUB paired-end shotgun data from 15 cities. The median percentage of unclassified reads for all CAMDA files was 44.92% (Table S1). Our analyses focused only on reads with annotated taxa. Principal coordinate analysis at normalized species abundance level with Bray-Curtis dissimilarity and heatmap visualization showed clustering of samples from Oceania, whereas other cities or continents overlapped one another in the first two dimensions (Fig. 3). Density plots of continents are reported in Figure S3. Better separation of continents was observed with the later dimensions. In order to predict locations of mystery samples from new origins, we modeled geographic coordinates using multivariate regression with Lasso regularization. We constructed a Lasso-regularized regression model at each taxonomic scale using the normalized abundance table of the corresponding taxa scale as feature data. To first confirm the potential of correctly predicting samples from a pre-trained city, we evaluate model performance using nested 10-fold CV. The model performance was the highest at the species level compared to that of genus and family in the 10-fold CV setting (Table 2). Scatterplots of true coordinates and the 10-fold CV predictions from species regression model showed a linear trend between predicted and true coordinates in Fig. 4a and b (r^2^ = 0.9258 for latitude and 0.8988 for longitude). In comparison, 10-fold nested CV performance of the Lasso regularized species classification model achieved a high accuracy level of 0.9457 (Fig. 4c).

**Table 2:** Nested 10-fold and leave-one-city-out cross validation performance for geographic coordinate prediction using multivariate regression with LASSO regularization

LASSO	EMP	lambda 1se	df	MSE 10-fold	MSE
					L1CO
**MetaSUB Shotgun**	species	1.9582	118	686	5303
	genus	1.5713	97	843	4671
	family	1.3495	86	1470	5650

### The challenge in prediction of new sources without previous samples

**Fig. 5:**
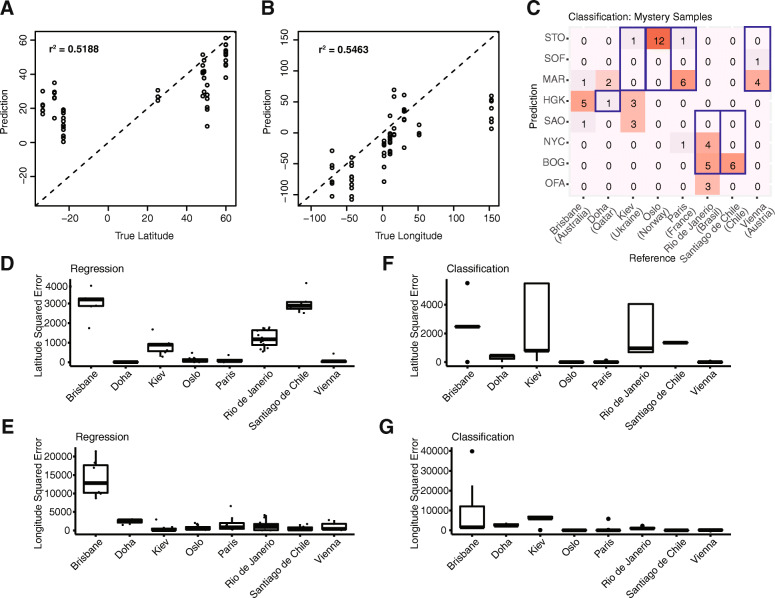
Source prediction of mystery samples from new cities using the species models. Latitude (**a**) and longitude (**b**) predictions from the multivariate regression model on species abundance data are plotted against the true geographic coordinates on the x-axis. Each data point represents a sample. The dashed line shows where predictions would be exactly correct. **c** Predictions from the classification model are illustrated in comparison to true sources. Each entry shows the number of samples predicted to be the corresponding city (row) and originally from the corresponding reference (column). Cities within the same continent with respect to reference are boxed in blue. Squared errors for latitude and longitude from the regression (**d**, **e**) and classification (**f**, **g**) are shown in boxplots for each city

To evaluate the prediction performance on samples from new origins, we conducted leave-1-city-out CV (Figure S4). Different from the 10-fold CV setting, the model performance was the highest at the genus level in the L1CO setting (Table 2). We proceeded with the analysis at the species level in the main manuscript, and attached the final MSEs from genus models in Table S3 for reference. The r^2^ values of the species regression model were 0.6031 and 0.2161 for latitude and longitude respectively. The L1CO MSE of the regression model is 8-fold the MSE of the nested 10-fold CV, which highlights the challenge in predicting new origins that have not been included in the model training. When predicting sources for the mystery data, the r^2^ values were 0.5188 and 0.5463 for latitude and longitude with the regression model (Fig. 5a, b). Contrary to our expectation, prediction performances on the mystery samples by species-level Lasso-regularized regression and classification models were comparable as indicated by MSE values (Table 3; Table S2). On the other hand, interestingly, the genus regression model had the best performance compared to all species and genus models, consistent with the L1CO assessment (Table S3). We also conducted random forest classification, a nonlinear approach, for comparison, but the classifier resulted in higher MSE values on mystery samples compared to Lasso-regularized models (Figure S5; Table S3). Overall, mystery samples from Brisbane had the highest prediction error in both Lasso-regularized models, despite having other Oceania samples from New Zealand as training data (Fig. 5d-g). The squared errors for samples from Kiev were significantly lower in the regression model than the classification model, indicating better performance by regression in these cases (one-sided Wilcoxon test, Benjamini-Hochberg adjusted *p* = 0.0284). On the other hand, predictions for Oslo, Paris and Santiago de Chile samples were better by classification (*p* = 0.0000, 0.0150, 0.0056). Despite overall comparable MSE values between the regression and classification models, we observed better prediction for some cites using either model.

**Table 3:** Prediction performance of species models on mystery (new) cities in the test dataset

Lasso-regularized	Paired & single end	Latitude MSE	Longitude MSE	M of total SE
Regression	No	1037.63	2629.85	3667.48
Classification	No	1143.58	2339.42	3483.00
Regression	Yes	1329.48	20,238.83	21,568.31
Classification	Yes	1468.31	7847.46	9315.76

As the classification model cannot accurately predict new origins at the level of longitude and latitude, we next evaluated the classification predictions in the context of continents. In L1CO CV setting, all samples from Auckland, Hamilton and Sofia were predicted to be on the same continent, while the rest of cities varied (Figure S4c). For mystery samples, all samples from Vienna, Santiago de Chile and Oslo were predicted to be cities within the same continent (Fig. 5c). Although Doha is considered to be in Asia, it is geographically closer to Marseille than to Hong Kong, and two of the three Doha samples were predicted to be from Marseille. Conversely, Kiev is within Europe and closer to European cities, but only one out of seven Kiev samples were predicted to be in Europe.

### Using prediction ambiguity to evaluate if a sample is from a new origin

**Fig. 6:**
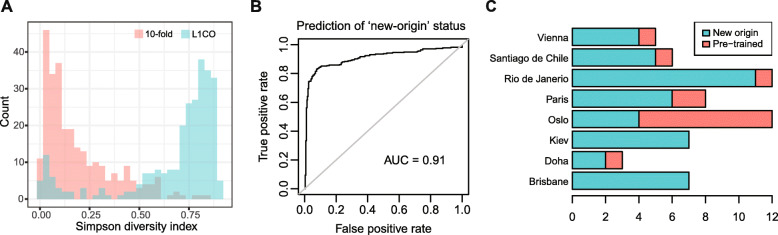
Ambiguity in classification prediction probabilities informs whether a sample is from a new origin. **a** The distributions of Simpson index on the class prediction probabilities of each sample based on 10-fold (red) and leave-one-city-out (blue) cross validation settings, which indicate the diversity pattern for samples from pre-trained or new origins, respectively. **b** The receiver operating characteristics curve of the Bayes classification model on predicting new-origin status using the Simpson index values computed through a leave-one-out design. **c** Prediction of new-origin status on mystery samples from new cities

Given the much lower prediction accuracy for samples from new origins, we rationalized that there are benefits to tag whether a new sample may be from a new origin in real life application when using a classification approach. We hypothesized that samples from new origins will have higher prediction ambiguities, as defined by the Simpson’s diversity index of a sample’s class probabilities. Specifically, we investigated the prediction ambiguity from the classification model on each leftout sample in both 10-fold and L1CO CV settings. As expected, leftout samples from new origins (in L1CO CV) have significantly higher ambiguity compared to leftout samples from pre-trained origins (in 10-fold CV; Wilcoxon test *p*-value = 6.4 × 10  ^− 58^; Fig. 6a). Based on Simpson’s values from both CV settings, we built a Bayes classifier with kernel density estimation on the Simpson’s values to classify whether a sample is from a new origin. The evaluation using leave-one-out CV reported an accuracy of 0.88 (sensitivity = 0.83; specificity = 0.92; Fig. 6b). Forty-six out of sixty mystery samples were correctly predicted to be from new origins using this binary classifier (sensitivity = 0.77; Fig. 6c). Oslo had the most samples wrongly predicted to be from pre-trained origins, whereas a subset of cities had one to two wrong predictions. We note that Oslo samples were predicted to be from Stockholm, which is also in northern Europe, and had low squared errors in the source classification model (Fig. 5 c, f, g). Hence, our new-origin classification model based on prediction ambiguity of the sample-source classification model indicated its potential to inform whether a mystery sample is from a new origin, which can serve as a flag to complement the lower prediction accuracy of samples from new origins.

### Inclusion of training data with different experimental protocols impacts model performance

Due to protocol differences in sequence lengths and single versus paired-end sequencing, we excluded the training data from the 16th city, SAC, provided by CAMDA in the main analyses above. To evaluate the effect of mixing data from different experimental protocols, we also examined the model performance with the inclusion of the SAC along with 15 other cities as training data (Table 3 and Figure S6). The heatmap and dimension reduction figure analogous to Fig. 3 are presented in Figures S6A-C. The MSEs of regression and classification models on the mystery samples increased 5.9- and 2.7-folds compared to models trained on 15 cities, respectively. Overall, the regression predictions resulted in a longitudinal shift away from the diagonal (Figure S6d). Notably, the longitudinal squared errors of Oslo samples increased substantially after incorporating SAC data for training in the classification model, while the squared errors increased for multiple cities in the regression model (Figure S6g and h). Specifically, the classification model predicted Oslo samples to be in Sacramento exclusively, and data from Oslo were all single-ended as Sacramento data (Figure S6f). These results indicated the impact of incorporating datasets with very different sequencing protocols.

## Discussion

Here we utilized MetaSUB and Boston Urban datasets provided by the CAMDA organizers to extract knowledge and elucidate factors that impacted the prediction of sample sources. Through the comparison between 16S amplicon and shotgun metagenomic sequencing data on the same Boston samples, we highlighted differences in detection sensitivity and normalized abundance between sequencing technologies as well as analytical tools and databases used. On predicting sample sources with MetaSUB data, we demonstrated the importance of using 10-fold CV to evaluate prediction performance on samples from pre-trained origins, and using leave-1-city-out CV to evaluate prediction performance on samples from new origins. The substantially higher prediction errors of the L1CO CV highlighted that predicting samples from new cities is much more challenging than samples from pre-trained cities. Our comparison of Lasso-regularized classification and regression approaches reported comparable MSE levels on mystery samples, and showed the benefits of either approach for predicting trained and new origins. As an extension of the source classification model, our use of prediction ambiguity based on the Simpson’s index allowed flagging of samples from a new origin. Lastly, we demonstrated reduced model performance when incorporating a dataset from a different experimental protocol as training data, highlighting the impact of heterogeneous experimental protocol.

Consistent with previous work comparing between 16S versus pair-end shotgun data [4–7], we reported variation in normalized abundance results between taxonomic classification tools and databases in addition to sequencing technologies using the Boston data. Our results further showed higher variation between technologies for taxa with lower abundance, and demonstrated that zero inflation in abundance is dependent on both experimental and analytical approaches. The zero inflation in 16S data compared to shotgun data by Kraken2 and Bracken is potentially due to high conservation of 16 s rRNA gene making finer taxonomic levels more difficult to identify [35]. Amplification biases attributed to use of the 16S marker gene have been previously described, and may have also contributed to variability between methods [36, 37]. Importantly, differential multiplexing in combination with other variation in sequencing methodologies employed by shotgun and 16S amplicon analysis can be another source of variation. On the other hand, the zero inflation observed in shotgun data by MetaPhlAn2 and its lower detection sensitivity is likely due to differences in the reference database used. To our knowledge, the default database used for the provided MetaPhlAn2 data was geared towards gut microbes, which may have resulted in information loss for the urban microbiome. Using the MetaSUB data, we demonstrated that heterogeneous experimental protocols used for sample collection and sequencing between cities can have a substantial influence on the prediction performance of new sources. The inclusion of single-end data from Sacramento as training data drastically reduced the performance of the model prediction on mystery samples. Short sequence reads are more challenging to taxonomically assign to a group, given the reduced amount of information available in each read. Alternative normalization and/or filtering approaches can be further evaluated and refined to account for the heterogeneity before downstream analyses [32, 33].

As reported in the 2018 CAMDA challenge [12, 15–17], our 10-fold CV results showed a strong potential to predict the source of samples from pre-trained origins using shotgun metagenomic data. We further demonstrated that the L1CO results highlighted the difficulty of predicting new origins without prior training samples, and confirmed L1CO to be a more realistic evaluation on the source prediction of mystery samples. The prediction performances between the regression and classification approaches were comparable with subsets of cities consistently predicted more accurately by either approach. While we presented species models in the main text, the genus regression model had the best prediction performance consistently in both L1CO CV and mystery samples. This may indicate that there is noise in the species data that the regression model is sensitive to, and that different modelling approaches may work better with data from different taxonomic ranks. Overall, the regression approach is limited by its assumption of linearity to coordinates, increased sensitivity to outlying coordinates, and the difficulty in interpreting feature importance along geographic coordinates. The classification approach can elucidate signatures in trained cities, but can only predict new origins to the closest cities at best. This limitation is addressed by our prediction ambiguity analysis to inform whether the sample is from a new origin, and may gradually be alleviated as samples from more origins are collected and pre-trained.

Here we used only normalized microbial taxonomic abundance from shotgun metagenomic data as the features to predict the sample source. Other covariates, such as micro-environment, seasons and city transportation connections [9, 38, 39] have been reported to impact bacterial abundance, and can be taken into account if the metadata were available. Moreover, given the advantage of shotgun data, further investigations of other types of features [40, 41] can be extended from our approach. These include using biologically-driven features such as functional pathways and antimicrobial resistance profiles [11, 17] and data-driven features such as counts of k-mers and genomic bins without annotation requirements [40, 42, 43], as alternative input variables. Such information can be incorporated into multifaceted analyses for sample source prediction through comparison of models with different feature types or integration of multi-layered feature sets. One key observation is the high proportion of unclassified sequences across many of the samples included in this analysis. Importantly, the continual updates in databases with incorporation of newly identified taxa will ultimately enhance the capacity to delineate signals between sources, whereas annotation independent information extraction such as k-mer counts may avoid missing out on unannotated taxa that differ in abundance between cities.

## Conclusions

In this work, we have highlighted the impact of sequencing approaches, taxonomic annotation tools, databases and heterogeneous protocols on result interpretation and model performance. We demonstrate the practical purpose of performance evaluation using 10-fold and leave-one-city-out cross validation for predicting pre-trained and new origins, respectively. The proposed Lasso-regularized multivariate regression provided a novel and alternative approach to source prediction with comparable performance to the classification approach. Due to the demonstrated challenge in predicting new origins without any metadata, we further provided a strategy to flag whether a sample is from a new origin for real life applications using the ambiguity of classification prediction. Overall, our work informs future metagenomics studies on the potential and challenges for source prediction using machine learning methods.

## Supplementary Information

**Additional file 1: Figure S1.** The world map labeled with training origins and mystery new origins. Mystery cities were labeled as question marks.

**Additional file 2: Figure S2.** Read count threshold evaluation using the Boston SG-KB and 16S data. Evaluation based on Boston-SG-KB data was conducted at varying minimum read count thresholds as colored in the legend. Each threshold is represented as a line in all figures. (A) Plot of feature/taxa counts in the y-axis versus sample counts in the x-axis satisfying the corresponding read count threshold. (B-D) The Pearson Correlation Coefficients between Boston-SG-KB data and Boston 16S data at varying taxa levels given that the corresponding read count threshold is satisfied in at least 1(B), 2(C), and 5(D) samples.

**Additional file 3: Figure S3.** Distribution of projected axes by continents. Density plots of samples organized in continent categories in the first four dimensions of the PCoA from Fig. 3b.

**Additional file 4: Figure S4.** Leave-one-city-out cross validation predictions from Lasso-regularized regression and classification compared to true locations. Latitude (A) and longitude (B) predictions from the multivariate regression model on species abundance data are plotted against the true geographic coordinates on the x-axis. Each data point represents a sample from the corresponding city, as indicated in the legend. The dashed line shows where predictions would be exactly correct. (C) Predictions from the classification model are illustrated in comparison to true sources. Each entry shows the number of samples predicted to be the corresponding city (row) and originally from the corresponding reference (column). As classification models can only assign new samples to pre-trained sources, diagonal counts are zero. Cities within the same continent are boxed in blue.

**Additional file 5: Figure S5.** Out-of-bag and mystery sample prediction performance using random forest classification algorithm. (A) Model performance as assessed from out-of-bag prediction. (B) Source prediction of mystery samples versus the reference. Cities within the same continent with respect to reference are boxed in blue. Squared errors for latitude (C) and longitude (D) are shown in boxplots for each city.

**Additional file 6: Figure S6.** Inclusion of training data from a heterogeneous sequencing protocol affects model performance. This figure is analogous to Figs. 3, S3 and 5, with the distinction of including single-end data from Sacramento into training. Figures (A-C) provide global visualizations of the samples using Bray-Curtis dissimilarity matrix. Latitude (D) and longitude (E) predictions from the multivariate regression model on species abundance data are plotted against the true geographic coordinates on the x-axis. Each data point represents a sample. The dashed line shows where predictions would be exactly correct. (F) Predictions from the classification model are illustrated in comparison to true sources. Each entry shows the number of samples predicted to be the corresponding city (row) and originally from the corresponding source (column). Cities within the same continent with respect to reference are boxed in blue. Squared errors for latitude and longitude from the regression (G, H) and classification (I, J) are shown in boxplots for each city.

**Additional file 7: Table S1.** Sequencing data information.

**Additional file 8: Table S2.** Mystery sample predictions.

**Additional file 9: Table S3.** Prediction performance of all reported models on mystery data.

## Data Availability

The scripts for the data processing and analyses are available at https://gitlab.cscscience.ca/nml/camda19-lassoreg. No experimental datasets were generated for the study. The datasets used are available through the CAMDA 2019 website (http://camda2019.bioinf.jku.at/doku.php/contest_dataset#metagenomic_forensics_challenge).

## Abbreviations

bp: Base Pair
CAMDA: Critical Assessment of Massive Data Analysis
CV: Cross Validation
L1CO: Leave-1-City-Out
MetaSUB: Metagenomics & Metadesign of Subways & Urban Biomes
MSE: Mean Squared Error
PCoA: Principal coordinate analysis
SG-KB: Organism abundance extracted by the Kraken2 and Bracken tools
SG-MP: Organism abundance provided by CAMDA using the MetaPhlAn2 tool
