# Supplementary figures and images for: Systematic evaluation of supervised machine learning for sample origin prediction using metagenomic sequencing data
Source: Biol Direct. 2020 Dec 10;15:29. doi: 10.1186/s13062-020-00287-y (PMC7731568; doi:10.1186/s13062-020-00287-y)

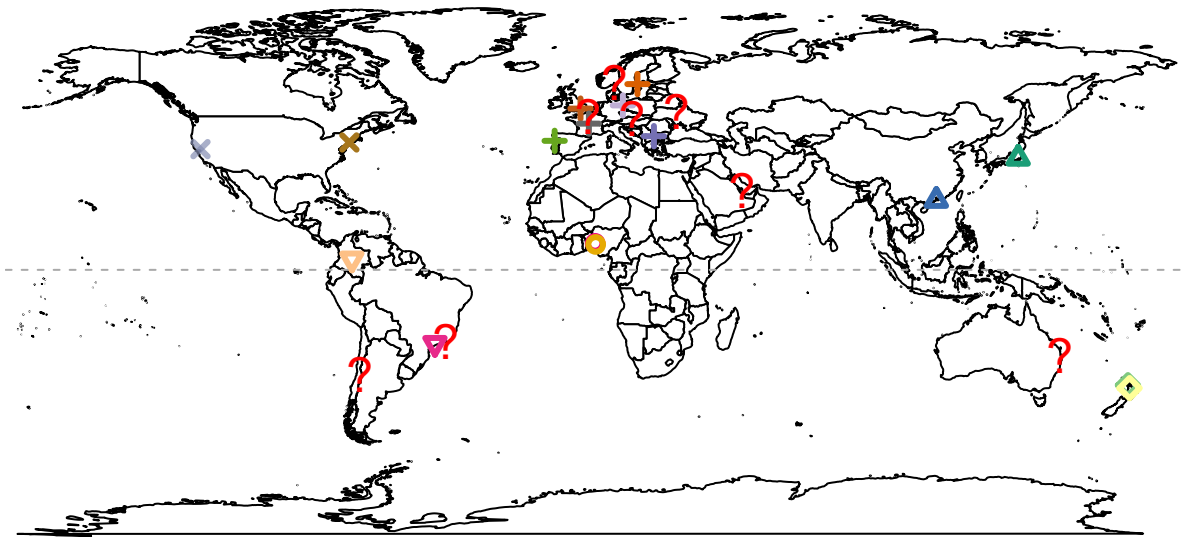

Supplement: Supplementary file 1 — Additional file 1: Figure S1. The world map labeled with training origins and mystery new origins. Mystery cities were labeled as question marks. [file 13062_2020_287_MOESM1_ESM.pdf]

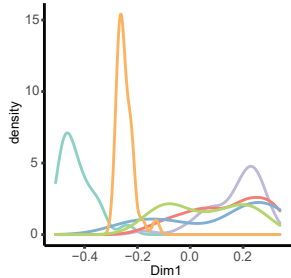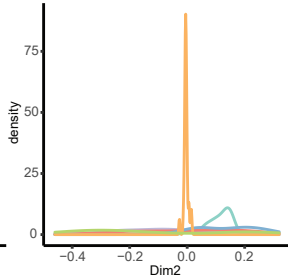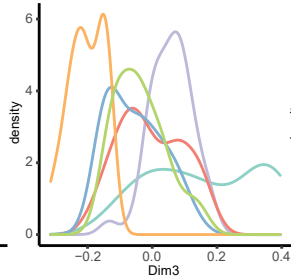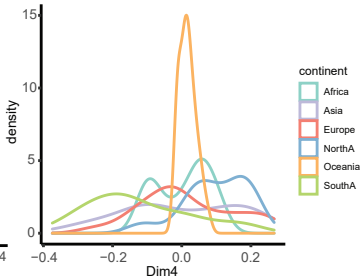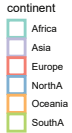

Supplement: Supplementary file 3 — Additional file 3: Figure S3. Distribution of projected axes by continents. Density plots of samples organized in continent categories in the first four dimensions of the PCoA from Fig. 3b. [file 13062_2020_287_MOESM3_ESM.pdf]

A

◆ AKL ◆ HAM + LON ○ OFA + SOF  
 + BER ▲ HGK + MAR + PXO + STO  
 ▼ BOG ● ILR × NYC ▼ SAO ▲ TOK

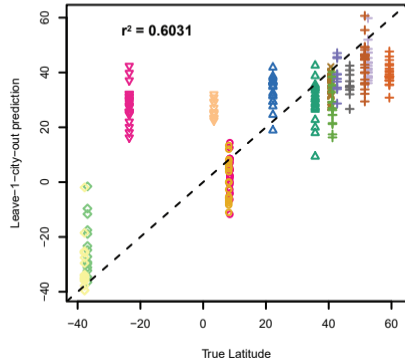

B

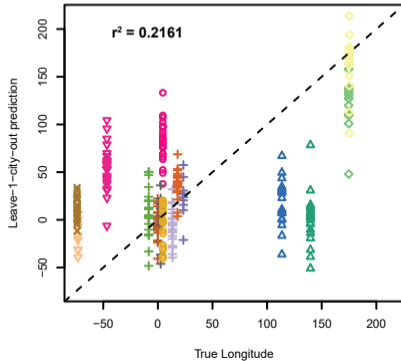

C

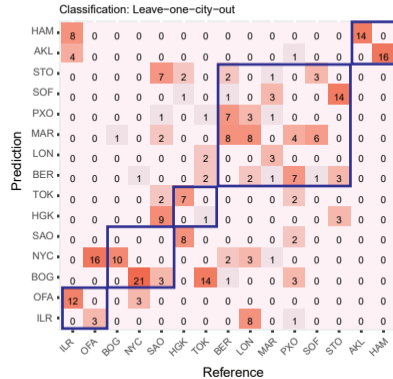

Supplement: Supplementary file 4 — Additional file 4: Figure S4. Leave-one-city-out cross validation predictions from Lasso-regularized regression and classification compared to true locations. Latitude (A) and longitude (B) predictions from the multivariate regression model on species abundance data are plotted against the true geographic coordinates on the x-axis. Each data point represents a sample from the corresponding city, as indicated in the legend. The dashed line shows where predictions would be exactly correct. (C) Predictions from the classification model are illustrated in comparison to true sources. Each entry shows the number of samples predicted to be the corresponding city (row) and originally from the corresponding reference (column). As classification models can only assign new samples to pre-trained sources, diagonal counts are zero. Cities within the same continent are boxed in blue. [file 13062_2020_287_MOESM4_ESM.pdf]

A

OOB seed 1 : Overall accuracy = 0.9601

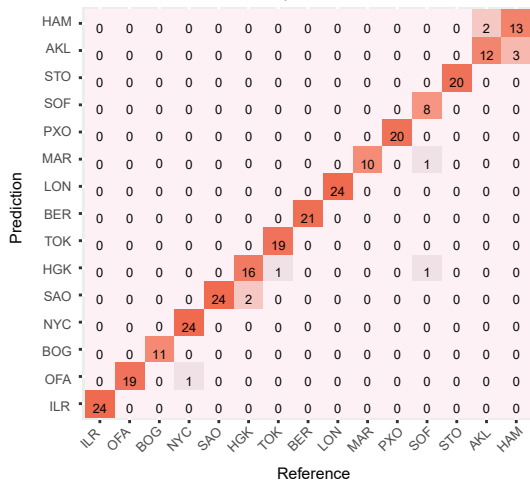

B

Classification: Mystery Samples

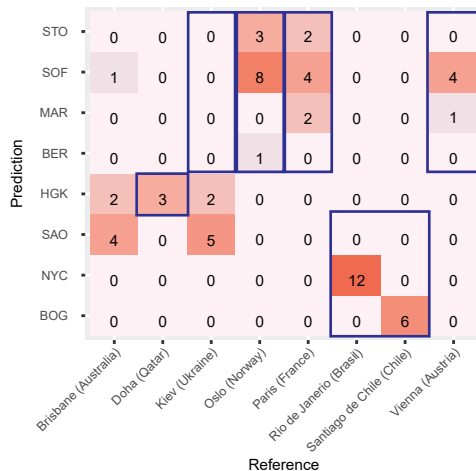

C

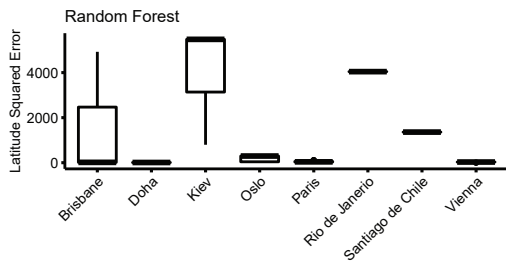

D

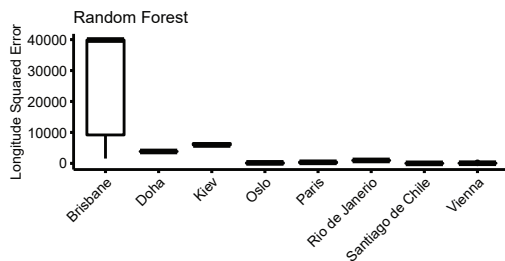

Supplement: Supplementary file 5 — Additional file 5: Figure S5. Out-of-bag and mystery sample prediction performance using random forest classification algorithm. (A) Model performance as assessed from out-of-bag prediction. (B) Source prediction of mystery samples versus the reference. Cities within the same continent with respect to reference are boxed in blue. Squared errors for latitude (C) and longitude (D) are shown in boxplots for each city. [file 13062_2020_287_MOESM5_ESM.pdf]

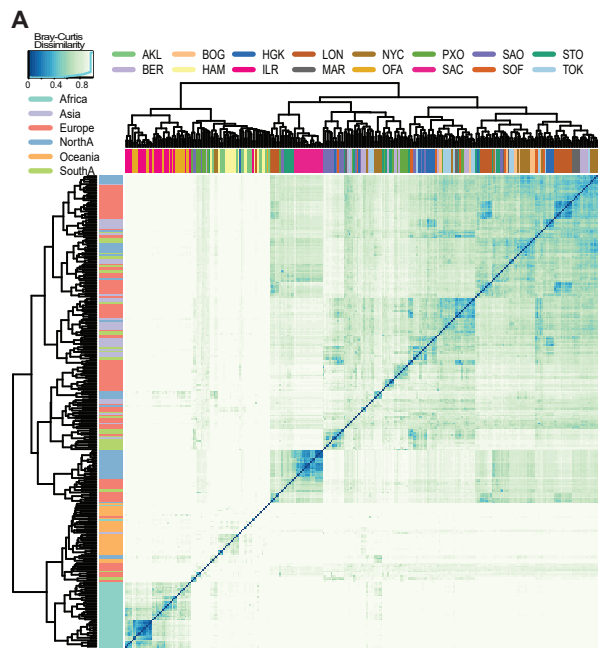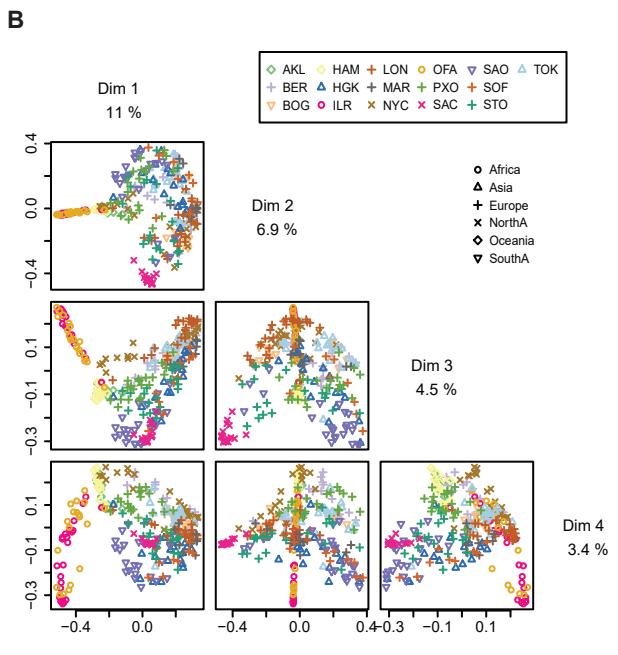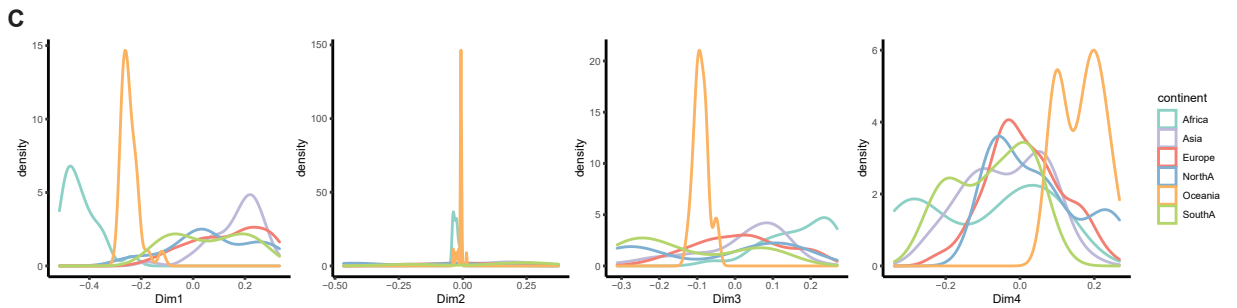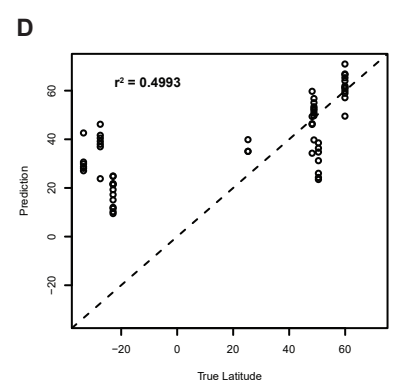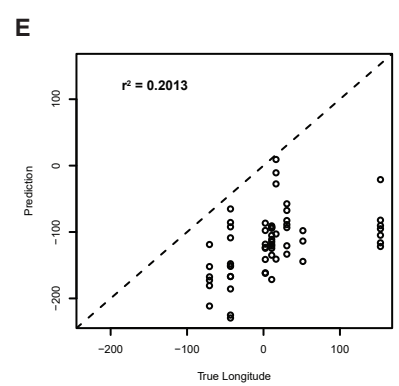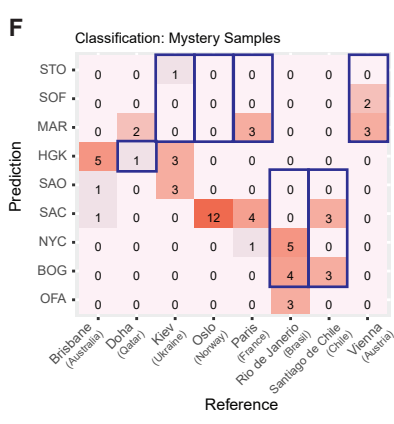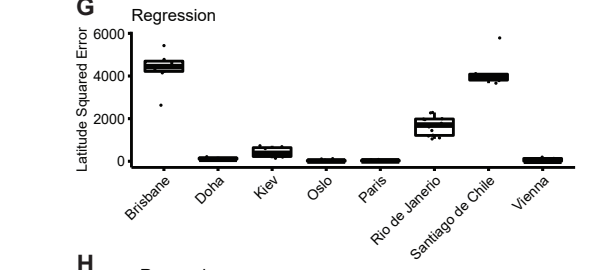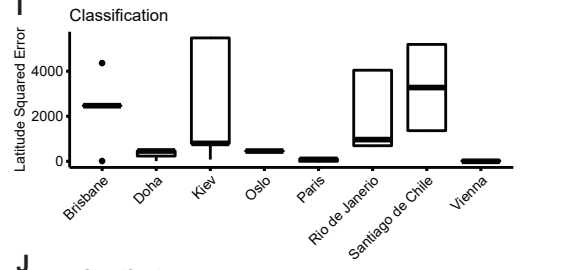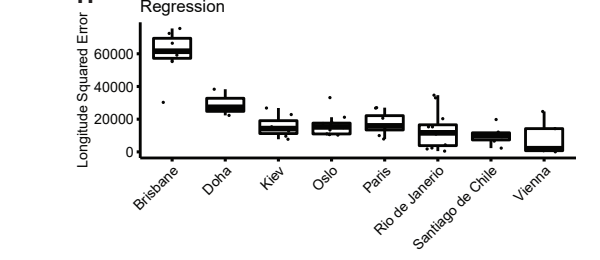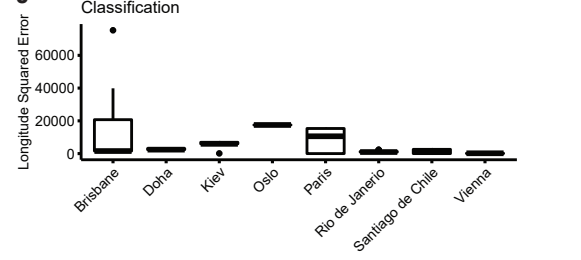

Supplement: Supplementary file 6 — Additional file 6: Figure S6. Inclusion of training data from a heterogeneous sequencing protocol affects model performance. This figure is analogous to Figs. 3, S3 and 5, with the distinction of including single-end data from Sacramento into training. Figures (A-C) provide global visualizations of the samples using Bray-Curtis dissimilarity matrix. Latitude (D) and longitude (E) predictions from the multivariate regression model on species abundance data are plotted against the true geographic coordinates on the x-axis. Each data point represents a sample. The dashed line shows where predictions would be exactly correct. (F) Predictions from the classification model are illustrated in comparison to true sources. Each entry shows the number of samples predicted to be the corresponding city (row) and originally from the corresponding source (column). Cities within the same continent with respect to reference are boxed in blue. Squared errors for latitude and longitude from the regression (G, H) and classification (I, J) are shown in boxplots for each city. [file 13062_2020_287_MOESM6_ESM.pdf]
