# Supplementary material for: Systematic evaluation of supervised machine learning for sample origin prediction using metagenomic sequencing data
Source: Biol Direct. 2020 Dec 10;15:29. doi: 10.1186/s13062-020-00287-y (PMC7731568; doi:10.1186/s13062-020-00287-y)
Supplement: Supplementary file 2 — Additional file 2: Figure S2. Read count threshold evaluation using the Boston SG-KB and 16S data. Evaluation based on Boston-SG-KB data was conducted at varying minimum read count thresholds as colored in the legend. Each threshold is represented as a line in all figures. (A) Plot of feature/taxa counts in the y-axis versus sample counts in the x-axis satisfying the corresponding read count threshold. (B-D) The Pearson Correlation Coefficients between Boston-SG-KB data and Boston 16S data at varying taxa levels given that the corresponding read count threshold is satisfied in at least 1(B), 2(C), and 5(D) samples. [file 13062_2020_287_MOESM2_ESM.pdf]

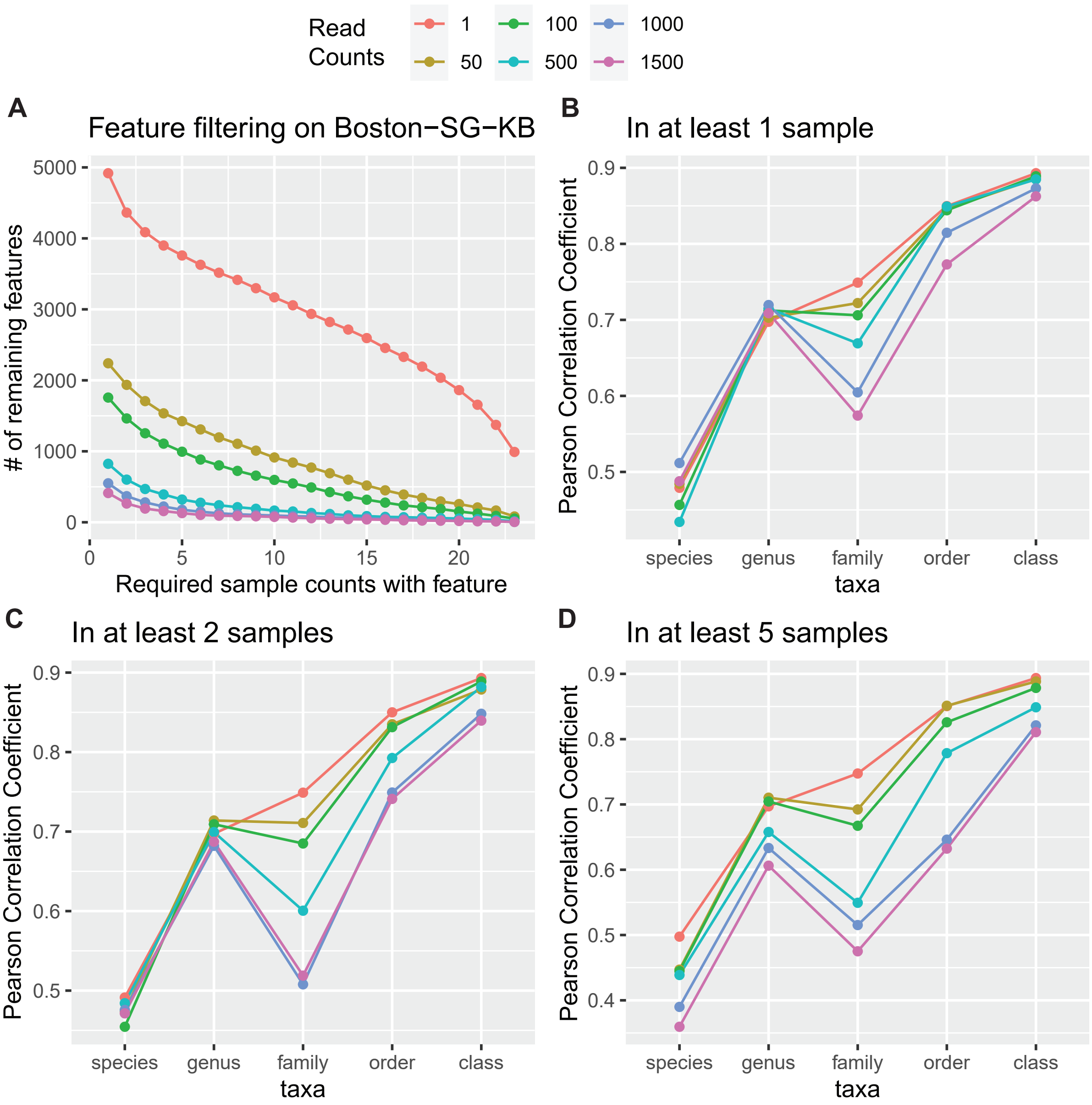
Read Counts
1
50
100
500
1000
1500
A
Feature filtering on Boston−SG−KB
# of remaining features
Required sample counts with feature
B
In at least 1 sample
Pearson Correlation Coefficient
taxa
C
In at least 2 samples
D
In at least 5 samples
species
genus
family
order
class
